# Developing and Examining the Effectiveness of a Cognitive Behavioral Therapy-Based Psychoeducation Practice for Reducing Obsessive-Compulsive Symptoms in Adolescents: A Mixed-Methods Study With a Turkish Sample

**DOI:** 10.3389/fpsyg.2022.805035

**Published:** 2022-03-07

**Authors:** Mustafa Kerim Şimşek, İsmail Seçer

**Affiliations:** ^1^Ministry of National Education, Yozgat, Turkey; ^2^Faculty of Education, Counseling and Guidance, Atatürk University, Erzurum, Turkey

**Keywords:** obsessive-compulsive symptoms, cognitive behavioral therapy, adolescent, psychoeducation, embedded mixed methods

## Abstract

This study developed a cognitive behavioral therapy (CBT)-based psychoeducation practice aimed at reducing obsessive-compulsive symptom levels in adolescents in Turkey and tested its effectiveness with a mixed-methods study. After the study was constructed as a pretest-posttest control group experimental application consisting of qualitative stages. The experimental application of the study was carried out with high school students in Turkey. In the sampling process, the schools, where the study will be carried out, were determined with the cluster sampling method. The experimental and control groups were formed with 30 students with high obsessive-compulsive disorder (OCD) symptoms from these schools, and the developed CBT-based psychoeducation practice was applied to the experimental group for 12 weeks. Quantitative data were collected through the “Child Version of the Obsessive-Compulsive Inventory,” and normality analysis, *t*-test for dependent samples, and Single Factor Analysis of Covariance were used. Qualitative data of the study were collected through document review, session evaluation forms, and semi-structured interview protocol; content and descriptive analysis techniques were used in the analysis. It was concluded that the developed CBT-based psychoeducation application was an effective approach in reducing OCD symptoms in the Turkish adolescent sample, except for the neutralizing dimension. It was also determined that the findings obtained from the analysis during the application and the interviews after the application are parallel with the findings obtained by quantitative methods, and the qualitative and quantitative findings adequately explained the quantitative documents findings.

## Introduction

Education and training processes can be shown as important life period thresholds in which acquisitions, such as adapting to social life, achieving academic progress and success, being beneficial to himself and his environment, acquiring a profession, and making free and independent decisions, are acquired for each individual forming the society (Caspi et al., 2005; Shaffer, 2008). It is stated that one of the periods in which the disruptions and possible problems in the acquisition of these skills in the education process are most intense during adolescence (Varela et al., 2017). Some adjustment problems caused by intense physical and emotional changes experienced in adolescence may cause disagreements in the relations of adolescents with their family and environment, trigger some psychological problems, decrease their academic achievement, and cause significant deterioration in social functioning (Derman, 2008). While these adjustment problems and mood changes caused by adolescence sometimes show themselves with behavioral problems, such as social withdrawal or anger in daily life, they can sometimes show themselves with symptoms of mental illness. Obsessive-compulsive disorder (OCD) and its symptoms can be shown as one of the symptoms of mental illness caused by adjustment problems (Valderhaug and Ivarsson, 2005; Coluccia et al., 2017).

OCD, which includes the adolescence period considered to be risky in terms of its symptoms (Öztürk and Uluşahin, 2011), is a mental disorder characterized by organized obsessions with compulsive thoughts, images, urges, or doubts and compulsions that involve rituals to reduce anxiety, control, or resist unwanted thoughts and avoidance (Abramowitz and Jacoby, 2015). The fact that the WHO considers OCD as one of the 10 diseases that cause the most disability in the individual points out that it is a serious disorder that has the power to directly affect the development of individuals (Beşiroǧlu and Aǧargün, 2006). OCD and its symptoms occur as a result of adjustment problems in adolescence and are also considered as an important risk factor in triggering other adjustment problems such as depression and anxiety (Hofer et al., 2018; Jones et al., 2018), peer bullying, truancy, and irritability (Sulkowski et al., 2018; Guzick et al., 2021; Veale and Willson, 2021).

The symptoms of OCD are not severe in the initial phase (Nikolajsen et al., 2011). In a study conducted in Turkey, the one-year incidence of OCD and its symptoms in adolescents was reported as 5.9% (Akbaba et al., 2010). In a study conducted by Abay et al. (2010) in a city center in Turkey, they found the prevalence of OCD in adolescents to be 1.4%. In another study conducted in Turkey, it was stated that OCD was detected in 2.7% of the patients who applied to child and adolescent psychiatry (Diler and Avci, 2002). When the symptoms are neglected or ignored, their course may worsen over time, turning into a disorder and making treatment more difficult (Mataix-Cols et al., 2008; Barton and Heyman, 2016). Symptoms can also trigger many other problems, such as low academic achievement, dislike of school, social isolation, peer conflicts, family conflicts, sleeping problems, suicidal thoughts, and life dissatisfaction (Lewin et al., 2013; Weidle et al., 2014; Huz et al., 2015; Reynolds et al., 2015). It is thought that raising awareness of obsessive-compulsive symptoms, which can cause the aforementioned problems and are shown as a risk in this context, will contribute to a healthier fulfillment of responsibilities in the social, academic, and personal lives of adolescents. In addition, considering the nature of the course of obsessive-compulsive symptoms that usually lead to the disorder (Freeman et al., 2007), it is important to make preventive and early interventions.

There was a belief that obsessive-compulsive symptoms were rare in children and adolescents compared to adults, and it was believed that the symptoms were harmless child rituals (Koşe, 2010). In recent years, increasing the awareness of OCD symptoms in children and adolescents and the rising trend in the number of studies on its prevalence has changed this belief and are considered as important developments in terms of early detection and intervention of symptoms. Studies on OCD and its symptoms state that the incidence in children and adolescents varies between 1 and 4% (Zohar, 1999; Altıntaş and Özçürümez, 2015; Jaisoorya et al., 2015; Politis et al., 2017; Saad et al., 2017). This result explains that OCD is diagnosed with at least one symptom. The findings show that obsessive-compulsive symptoms are an important risk factor for OCD, and early detection and intervention are important. It has been stated that OCD treatment interventions in adolescents focus on the disorder rather than the symptoms, and one of the most effective treatment methods is cognitive behavioral therapy (CBT) with medication (Pediatric Obsessive-Compulsive Disorder Treatment Study [POTS] Team, 2004; Franklin et al., 2011; Piacentini et al., 2011). Although the number of treatment interventions related to OCD is high, it is noteworthy that studies on intervention and prevention approaches for its symptoms are relatively few (Freeston et al., 1997; Wootton, 2016). In Turkey, there is no psychoeducation practice based on CBT regarding OCD symptoms in adolescents. This shows that there is a need for a systematic intervention program that can be used for preventive interventions for OCD symptoms, which can negatively affect many areas of the life of an adolescent. At this point, it is thought that a study on the development and testing of psychoeducational practice including CBT methods in order to recognize obsessive-compulsive symptoms and to intervene early in the Turkish adolescent sample will make a significant contribution to the field and all stakeholders working in the field of mental health. In this respect, it is thought that it will fill this gap in the field with its important original value. It is also important that the mixed methods research was used in both the development and testing of the intervention program in this process, in terms of reflecting another unique aspect of the research and contributing to the mixed method research literature. In this context, this study developed and tested the effectiveness of a CBT-based psychoeducation practice aimed at reducing OCD symptoms and preventing the progression of symptoms to a disorder in a Turkish adolescent sample. The research is based on the following questions and hypotheses.

**Research question:** “How do qualitative results from document analysis and interview data on OCD and its symptoms in adolescents help in developing an intervention program for OCD symptoms and explaining the program’s outcomes by testing its effects?”

**Research hypotheses:**

1. The application of the psychoeducational program developed on the basis of CBT is an effective intervention approach in reducing OCD symptoms in adolescents.
2. The OCD symptoms of the adolescents participating in the CBT -based psychoeducation program differ significantly compared to the non-participating group.

## Materials and Methods

### Research Design

**FIGURE 1:**
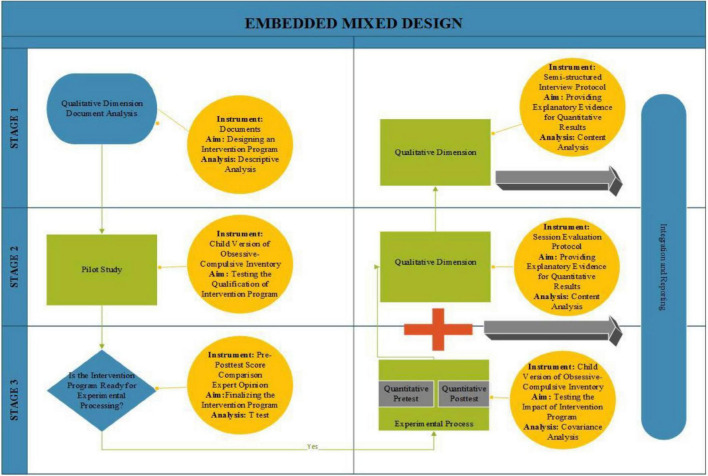
Research design diagram.

A mixed methods research, in which both quantitative and qualitative research approaches are used at certain stages, was used (Creswell, 2019). An embedded mixed design, one of the mixed method approach designs, was used to interpret quantitative and qualitative results and to obtain more detailed inferences about the quantitative results (Creswell and Plano Clark, 2018). In the quantitative phase of the research, the experimental design with the pretest post-test control group (Büyüköztürk et al., 2013; Gürbüz and Şahin, 2016) was used, and the phenomenological approach was used in the qualitative phase. In the quantitative stage, the experimental design with the pretest post-test control group was used to test the developed intervention program on the experimental group and to determine whether the application had an effect on the results. In the qualitative stage, semi-structured interviews were conducted with the participants in the experimental study to describe in detail the phenomenon experienced after the intervention (Creswell, 2016). Within the scope of the research, document analysis technique, which is one of the data collection techniques commonly used in qualitative studies, was used to collect data before, during, and after phenomenology and to develop a psychoeducation program by determining the general structure of OCD symptoms (Gürbüz and Şahin, 2016). One of the reasons for the mixed methods research of this study is the weakness of the qualitative approach toward generalization, which allows it to be used with the generalizable nature of the quantitative approach. Another reason is that it enables researchers to obtain complementary results by detailing the findings obtained from quantitative data with qualitative content analysis. Another important reason is that it has a development aspect to obtain more effective and clarified results by adding qualitative research method data to the findings obtained from the quantitative method (Plano Clark and Ivankova, 2016). In the research process, quantitative and qualitative data types played an equally important role in answering the research questions. A visualized diagram of the research design is shown in Figure 1.

### Study Group, Participants, and Data Sources of the Study

The target population of the study is high school students from Yozgat City Center with high OCD symptoms. First, the experimental and control groups were determined among the schools in the target population, and the two schools where the application would be carried out were selected by simple random sampling method. Then, the participants with high OCD symptoms to be included in the experimental and control groups were determined by criterion sampling. In order to determine the study group within the scope of the experimental application, “the explanatory sequential design,” specific to mixed-methods research, was used, and the larger sample used in the quantitative dimension was limited to the qualitative dimension. Then the sampling frame was created (Teddlie and Tashakkori, 2015). It is stated that randomization is an important condition in experimental studies to be able to state that the changes observed in the dependent variable are due to the independent variable (Gürbüz and Şahin, 2016). For this purpose, in the quantitative dimension of the study, a total of 15 people, 9 females and 6 males, with high OCD symptoms were included in the experimental group, and a total of 15 people, 8 females and 7 males, were included in the control group by drawing lots using simple random sampling method. For the design of the psychoeducation program, the subsampling was applied to the document analysis, and in this context, theses open to access at the National Thesis Center of the Council of Higher Education and published studies accessible in Google Academic, ScienceDirect, PsycINFO, and ULAKBİM databases between 2010 and 2019 that can be accessed with the terms “*obsessive-compulsive disorder in adolescents”* and/or *“cognitive behavioral therapy*” and the keywords related to these concepts, and as a result, 16 theses and 52 articles were determined as the data sources of the document review dimension.

### Validity and Reliability of the Study

To ensure internal validity in this study, the participants, who formed the study group, were selected from students with high OCD symptoms through randomization (unbiased selection). The experimental and control groups of the study were formed from 2 different schools through unbiased selection, and their assignments to groups 1 and 2 were made by drawing lots. In order to avoid the maturation effect that might affect the internal validity, the sessions were designed as 12 sessions, not exceeding 90 min. The same measurement tools were used in the experimental and control groups to detect OCD symptoms and to measure the effect of the intervention strategy. For the statistical validity of the study, it was examined whether the data met the parametric test conditions, and it was determined that the parametric conditions were met by using statistical methods. In addition, covariance analysis, one of the statistical methods, was used to eliminate the possible effect of the pre-test. In order to ensure the construct validity of the study, the theoretical and practical framework of the concept to be intervened (OCD symptoms) was created and analyzed in the document analysis for the designed intervention program. In order to ensure external validity, the experimental and control groups were composed of 15 people, and the intervention was carried out at school and on a voluntary basis in order to provide an environment in which the participants could be generalized (Teddlie and Tashakkori, 2015). Test fidelity was observed in order to increase the reliability, and none of the items changed, which could affect the structure of the measurement tools used.

To ensure consistency for the qualitative dimension, no changes were made in the intervention program developed during the research process, and the process was recorded (Teddlie and Tashakkori, 2015). Data source triangulation was used to ensure the credibility of the findings, and the triangulation strategy (Creswell, 2016) was used to provide evidence for the information obtained using multiple and different data sources. In order to ensure the transferability principle of the research, the research process was described in detail, and the narrative was enriched by using qualitative and quantitative data. To ensure the confirmability principle in the research process, the method and data collection process were described in detail, audio recordings were taken for qualitative data, transcriptions were done, and these were supervised by two experts (Lincoln and Guba, 1985).

### Measures

#### Child Version of OCI

The child version of OCI developed by Foa et al. (2010) to determine the level of obsessive-compulsive symptoms in children and adolescents was adapted to Turkish culture by Seçer (2014). Confirmatory factor analysis was used for model fit in the adaptation process of the scale, and it was found that the factor and item structure of the scale, consisting of six sub-dimensions, in its original form showed good fit on the Turkish sample. As a result of construct validity: fit index values were found as (**χ^2^/**SD = 1.69, RMR = 0.046, SRMR = 0.048, CFI = 0.98, RMSEA = 0.046). Internal consistency and split-half validity were examined for the reliability of the scale, and the Cronbach’s alpha coefficient of the scale was found to be 0.86 and the split-half validity was 0.82.

#### Semi-Structured Interview Protocol

Two different semi-structured interview protocols were developed by the researchers. The first protocol was used to confirm the OCD symptom scores before the intervention program with 15 participants determined as the experimental group, and the second protocol was used to evaluate the program after the intervention program and to explain the quantitative data obtained in more detail. Necessary corrections were made by taking expert opinion for both protocols.

#### Session Evaluation Protocol

A session evaluation protocol was developed to obtain qualitative data from the participants during the experimental process. With the participant evaluation protocol, opinions about the sessions were collected through written evaluations. While developing the session evaluation protocol, expert opinion was used and necessary corrections were made.

#### The Process and Application

The child version of OCI was applied to the students in two schools, which were determined in order to carry out the experimental process with the designed intervention program, and the data were analyzed. Semi-structured interviews were conducted with students with OCD symptoms to be included in the experimental group as a result of the analyses. The families of the students who volunteered to participate in the intervention program among the students who met the specified criteria were reached and informed with a consent form, and necessary permissions were obtained from their families. With the determination of the experimental and control groups, a 12-week psychoeducation intervention was applied to the experimental group between “October 2019 and January 2020” and no activity was applied to the control group. During the research process, the experimental group participants and the session coordinator filled out the session evaluation protocols, and when the process was completed, a post-test was applied to the participants and semi-structured interviews were conducted with the experimental group.

In the data analysis process of the research, content and descriptive analysis techniques were used in order to analyze the data collected in three different time periods: documents related to the process of creating the first intervention program, during the intervention program, and semi-structured interviews after the intervention program (Yıldırım and Şimşek, 2016). Before and after the experimental application, measurements were made using the child version of OCI. Quantitative data analysis processes were carried out using the SPSS 22.0 package software for Windows. First, analyses of homogeneity of variances, normal distribution and extreme values were performed to determine whether the data were suitable for parametric test conditions. On condition that the parametric conditions were met, first, a *t*-test was performed for independent samples to determine whether the pre-test scores of the experimental and control groups were equal. Second, Single Factor Analysis of Covariance (ANCOVA) was conducted to determine whether the post-test scores of the experimental and control groups showed a significant difference after the experimental procedure.

#### Development of the Psychoeducation Program

In this study, a systematic and intervention-oriented psychoeducation program based on CBT was developed in order to reduce the OCD symptoms of adolescents. The program is based on psychoeducation in terms of its content and is intended to have curative effects. The document analysis method was used in the development of the program. Document analysis has been expressed as a systematic procedure carried out to review and evaluate printed and/or electronic materials (Bowen, 2009). With the document analysis, the concepts that were emphasized more and analyzed in studies on OCD and its symptoms between 2010 and 2019 were gathered under four themes: “Cause and symptoms,” “Cognitive and behavioral processes,” “Cognitive behavioral intervention methods,” and “Recurrence prevention strategies.” In order to obtain detailed information about the determined themes, to present the basic components of the psychoeducational program’s contents, and to design activity examples, “Variables addressed regarding cognitive behavioral interventions” were determined, “variables that are negatively and positively related to OCD and its symptoms” were discussed, and “variables with predictive effects on OCD and its symptoms” were determined, and the contents of the CBT-based psychoeducation program were designed. A pilot study was conducted in order to evaluate the quality of the psychoeducation program, whose content was designed, in terms of gaining the determined target behaviors and whether the time determined for each session was sufficient. Considering the situations mentioned as a result of the application, reorganizations were made regarding the relevance and timing of the activities. In addition, in order to compare the pretest, posttest and follow-up tests of OCD symptom scores on a group with high OCD symptoms related to the pilot application, a single factor ANOVA test was applied for dependent groups through the SPSS 22.0 package software for Windows. As a result, it was determined that there was a decrease in the OCD symptom scores of the participants compared to the pre-program, and it was determined that the reduction in OCD symptoms was maintained as a result of the 3-month follow-up [*F*(2, 18) = 18.931, *p* < 0.01]. The relevant sessions of the developed psychoeducation program are briefly presented in Table 1.

**TABLE 1:** Psychoeducation program contents.

Sessions	Purpose
Session 1	It is aimed that the group members get to know each other and the group leader, build trust among the members and know the rules to be followed in the group.
Session 2	It is aimed to give information about obsessions and compulsions, what their causes may be, their types and the false facts in dealing with them.
Session 3	It is aimed to recognize emotions, to realize what kind of schemes are used when interpreting emotions, and to show how emotions and obsessions affect each other.
Session 4	It is aimed to introduce “Automatic Thoughts” in order to show how effective dysfunctional thoughts are in OCD symptoms and to show the effect of these automatic thoughts in creating vicious circles that can occur in obsessions.
Session 5	It is aimed to evaluate the “Automatic Thoughts” of the group members and to examine how they interpret these thoughts.
Session 6	It is aimed to talk about cognitive models related to OCD symptoms and to enable members to reevaluate their thoughts within the scope of A-B-C-D model.
Session 7	It is aimed to evaluate how group members reinterpret their automatic thoughts according to the “Thought Registration Form” and to discuss the functionality of the techniques.
Session 8	It is aimed to make the group members realize the emotional changes by making balanced evaluations and to draw a new framework for the interpretations.
Session 9	It is aimed to show the group members that it is possible to make the necessary planning for behavioral intervention for their obsessions and make them ineffective and manageable over time by facing them.
Session 10	It is aimed to evaluate the confrontation ladder created by the group members for the disturbing situations related to their obsessions and to complete the missing parts by discussing them.
Session 11	It is aimed to monitor how/how much the group members are able to face their obsessions by using the “Exposure and Response Prevention” method and the “Confrontation Ladder” and to talk about alternatives that can be developed to the difficult points.
Session 12	It is aimed to talk about what can be done for the continuity and inclusion of the acquisitions gained from the past sessions in life.

## Results

In line with the mixed method question of the research *“How do qualitative results from document analysis and interview data on OCD and its symptoms in adolescents help develop an intervention program for OCD symptoms and test its effects to explain the program’s outcomes?”* the effect of the CBT-based OCD symptoms psychoeducation program on adolescents’ OCD symptoms was tested with the findings obtained from three different methods: experimental method, document analysis, and interview, within the scope of mixed methods research.

One-way analysis of covariance (ANCOVA) was conducted in order to examine what kind of differences the psychoeducation practice carried out within the scope of *“Psychoeducation program application developed on the basis of cognitive behavioral therapy is an effective intervention approach in reducing OCD symptoms in adolescents,”* the first quantitative hypothesis of the research, provided in the experimental and control groups. The condition of homogeneity of regression trends, which is the prerequisite for analysis of covariance before analysis was provided for doubting/checking [*F*(1, 22) = 0.808, *p* > 0.05], for obsessing [*F*(1, 22) = 1.298, *p* > 0.05], for washing [*F*(1, 22) = 0.939, *p* > 0.05], for ordering [*F*(1, 22) = 1.977, *p* > 0.05], and for mental neutralizing [*F*(1, 22) = 0.027, *p* > 0.05] from OCD symptom dimensions, and since the regression trends could not be matched for hoarding [*F*(1, 22) = 7.919, *p* < 0.05], *t*-test was applied for dependent samples to determine the significance of the change. After providing an important assumption of the analysis of covariance, the post-test mean scores of the OCD symptom dimensions were adjusted to determine whether the experimental group had significantly better improvements in OCD symptoms than the control group. It was determined that the change obtained as a result of the relevant corrections was significant in favor of the experimental group. The findings regarding the corrected post-test mean scores of the experimental and control groups are given in Table 2:

**TABLE 2:** Findings regarding the corrected post-test mean scores of the experimental and control groups.

Doubting/Checking	Group	n	Mean	Corrected mean
Post-test	Experimental	10	5.10	5.129
	Control	13	13.15	13.131

**Obsessing**	**Group**	**n**	**Mean**	**Corrected mean**

Post-test	Experimental	10	4.50	4.573
	Control	13	11.61	11.788

**Washing**	**Group**	**n**	**Mean**	**Corrected mean**

Post-test	Experimental	10	2.40	2.787
	Control	13	7.69	7.218

**Ordering**	**Group**	**n**	**Mean**	**Corrected mean**

Post-test	Experimental	10	2.90	2.46
	Control	13	6.00	6.09

**Neutralizing**	**Group**	**n**	**Mean**	**Corrected mean**

Post-test	Experimental	10	1.70	1.371
	Control	13	4.92	5.112

Following the findings in Table 2, the analysis was continued by keeping the pre-test scores under control to obtain information on whether there was a significant difference between the corrected annotated values. As a result of the relevant analysis, it was concluded that the CBT-based OCD psychoeducation application applied to adolescents provided significant reductions in OCD symptoms other than neutralization of the participants in the experimental group. In Table 3, 6 different dimensions of OCD symptoms are considered together and the relevant analysis results for each dimension are given. In the last part, considering the fact that the research was conducted with mixed-methods research, each finding was integrated by giving quantitative and qualitative data together.

**TABLE 3:** Analysis results on obsessive-compulsive disorder (OCD) symptom dimensions.

Source	Sum of squares	*SD*	Mean squares	*F*	*p*	η^2^
Corrected model	529.576	2	264.788	17.214	0.000	633
Intercept	26.263	1	26.263	1.707	0.206	079
Doubting/Checking pre-test	162.951	1	162.951	10.594	004	346
Group	361.924	1	361.924	23.529	0.000	541
Error	307.642	20	15.382			
Total	2980.000	23				
Total error	837.217	22				

**Source**	**Sum of squares**	***SD***	**Mean squares**	***F***	***p***	**η^2^**

Corrected model	296.588	2	148.294	16.194	0.000	618
Intercept	16.762	1	16.762	1.830	191	084
Obsessing pre-test	20.226	1	20.226	9.026	0.031	416
Group	296.466	1	296.466	0.32.374	0.000	618
Error	183.151	20	9.158			
Total	2150.000	23				
Total error	479.739	22				

**Hoarding**	**Measurement**	**N**	**X**	**SS**	***t***	***p***

	Pretest	10	7.10	2.46	6.651	0.000
	Post-test	10	1.40	1.89		

**Source**	**Sum of squares**	***SD***	**Mean squares**	***F***	***P***	**η^2^**

Corrected model	233.894	2	116.947	33.613	0.000	771
Intercept	10.704	1	10.704	3.076	095	133
Washing pre-test	75.585	1	75.585	21.725	0.000	521
Group	100.433	1	100.433	28.866	0.000	591
Error	69.585	20	3.479			
Total	972.000	23				
Total error	303.478	22				

**Source**	**Sum of squares**	***SD***	**Mean squares**	***F***	***P***	**η^2^**

Corrected model	83.905	2	41.953	7.405	004	425
Intercept	496	1	496	087	770	004
Ordering pre-test	29.588	1	29.588	5.222	033	207
Group	68.562	1	68.562	12.101	002	377
Error	113.312	20	5.666			
Total	695.000	23				
Total error	197.217	22				

**Source**	**Sum of squares**	***SD***	**Mean squares**	***F***	***P***	**η^2^**

Corrected model	70.408	2	35.204	4.364	027	304
Intercept	4.486	1	4.486	556	464	027
Neutralizing pre-test	11.692	1	11.692	1.449	243	0.068
Group	70.041	1	70.041	8.683	008	303
Error	161.331	20	8.067			
Total	517.000	23				
Total error	231.739	22				

It can be seen in Table 3 that the CBT-based OCD psychoeducation application applied to adolescents resulted in a significant decrease *in the doubting/checking* symptoms of the participants in the experimental group [*F*(1, 22) = 10.594, *p* < 0.05, η^2^ = 0.346]. The results of the Bonferroni test also showed that the results of the post-test were in favor of the experimental group.

It has been seen that the qualitative findings obtained through the session evaluation protocol and interview method during and after the experimental application explain and support the result of the experimental process. In the analysis of the documents collected from the participants during the sessions, *“I realized that I could achieve something”* statement of **Participant (P)3** and *“Most importantly, instead of running away from my obsessions, I learned that my life will be easier when I go above them and how to do it in 3 steps”* statement of **P5**, and the statements of the participants from the interviews after the application such as *“I don’t even check the stove after all, I mean maybe once”* by **P1** and *“I’ve started doing some stuff. I’ve started not checking my locker, once you have said that you should try something, I left the dorm leaving my keys there. I told myself that I can do things”* by **P2** among the participants with high doubting/checking symptom levels indicate that the program reached its goal in the doubting/checking dimension.

In Table 3, the CBT-based OCD psychoeducation application applied to adolescents resulted in a significant decrease *in the obsessing* symptoms of the participants in the experimental group [*F*(1, 22) = 9.026, *p* < 0.05, η^2^ = 0.416]. The results of the Bonferroni test also showed that the results of the post-test were in favor of the experimental group.

The qualitative findings obtained through the session evaluation protocol and interviews during and after the experimental application explain and support the result of the experimental process. In the analysis of the documents collected from the participants during the sessions, the statements of the participants with high levels of obsessions such as *“I learned the things I need to do to get rid of dysfunctional thoughts”* by **P7**, *“I think I’ve made my way up the ladder of confrontation so far”* by **P9**, and the statements of the participants in the findings obtained from the interviews after the experimental application such as *“For example, I had bad thoughts. I was very upset about everything; I was very worried about everything. It also affected my sleep a lot. It’s not like that anymore, I can even go to bed very early. Generally, I used to have bad thoughts about my friends. I’m not sad like I used to, I’m happier”* by **P8** and *“Because of my obsessions, I was only studying mathematics for university exams before the interviews. For example, 3 or 4 hours at home, but it was not very productive. I was solving the same problem, the same question over and over again, and the next day, I was solving two sources, for example, again. I was erasing and solving again, I was watching videos again and again”* by **P3** show that the program reached its goal in the obsessing dimension.

In Table 3, it is seen that the post-test mean scores of the *hoarding* sub-dimension of the participants in the experimental group of the CBT-based OCD psychoeducation application applied to adolescents changed significantly compared to the pre-test mean scores [*t*(9) = 6.651, *p* < 0.00]. In addition, the pre-test arithmetic averages of the participants are (= 7.10, *SD* = 2.46) and post-test arithmetic means are (= 1.40, *SD* = 1.89).

The qualitative findings obtained through the session evaluation protocol and interviews during and after the experimental application explain and support the result of the experimental process. In the analysis of the documents collected from the participants during the sessions, the statements of the participants with high levels of hoarding such as *“I learned to move slowly, with small and firm steps”* by **P1** and *“I realized that my obsessions sabotaged me”* by **P8**, and the statements of the participants in the findings obtained from the interviews after the experimental application such as *“I started talking to people while walking on the street, this is a pretty big change for me. Even my friends noticed that lately, Nisa is a close friend of mine, she was very uncomfortable with my gelatin collection and now she can tell me her feelings directly.”* by **P7** show that the program reached its goal in the hoarding dimension.

In Table 3, the CBT-based OCD psychoeducation application applied to adolescents resulted in a significant decrease in the *washing* symptoms of the participants in the experimental group [*F*(1, 22) = 21.725, *p* < 0.05, η^2^ = 0.521]. The results of the Bonferroni test also showed that the results of the post-test were in favor of the experimental group.

The qualitative findings obtained through the session evaluation protocol and interviews during and after the experimental application explain and support the result of the experimental process. In the analysis of the documents collected from the participants during the sessions, the statements of the participants with high levels of washing such as *“I learned that I cannot do everything perfectly”* by **P6** and *“In today’s session, I learned that we should face our obsessions rather than escape from them. I learned that we can deal with these.”* by **P10**, and the statements of the participants in the findings obtained from the interviews after the experimental application such as *“When I took off my socks, I always put water on my feet before going to bed. I don’t pay that much attention now. Also, my teacher, I was washing my hands all the time. My hands were dry, now a little less”* by **P1** and *“I had to study but instead I was cleaning and it was taking a long time. I had less time to study, but now it’s not like that, for example, I can finish my cleaning early and study immediately, it is good in terms of time”* by **P9** show that the program reached its goal in the washing dimension.

In Table 3, the CBT-based OCD psychoeducation application applied to adolescents resulted in a significant decrease in the *ordering* symptoms of the participants in the experimental group [*F*(1, 22) = 5.22, *p* < 0.05, η^2^ = 0.207). The results of the Bonferroni test also showed that the results of the post-test were in favor of the experimental group.

The qualitative findings obtained through the session evaluation protocol and interviews during and after the experimental application explain and support the result of the experimental process. In the analysis of the documents collected from the participants during the sessions, it was determined that there was only one participant with a high level of ordering symptom. The statement *“I think I have progressed on the ladder of confrontation so far”* quoted from **P9** and the findings obtained as a result of the interview made after the experimental application show that the statement of **P9** *“At first, I used to think that it wouldn’t go away, it was OK, but later on I realized how serious it was. Great troubles would await me in the future. As you see it go away and as the sessions progresses, you feel more comfortable. You have confidence, you don’t do those things but they don’t bother you as well”* shows that the program has achieved its goals of raising awareness about regular symptom levels and by reinforcing this awareness with confrontation studies.

In Table 3, the CBT-based OCD psychoeducation application applied to adolescents resulted in a significant decrease in the *neutralizing* symptoms of the participants in the experimental group [*F*(1, 22) = 1.449, *p* < 0.05, η^2^ = 0.068]. In the qualitative findings obtained during and after the experimental application, no expressions specific to the mental neutralizing dimension were found.

Within the scope of the second quantitative hypothesis of the study, *“The OCD symptoms of the adolescents participating in the cognitive behavioral therapy-based psychoeducation program differ significantly compared to the non-participating group,”* the total OCD symptom scores were examined. In the process, covariance analysis was used for OCD total symptom scores [*F*(1, 22) = 0.621, *p* > 0.05] by providing the prerequisite of covariance analysis, the congruence of regression trends. To determine whether the experimental group had significantly better improvements in OCD symptoms than the control group, the OCD total posttest mean scores were adjusted. It was determined that the change obtained as a result of the relevant corrections was significant in favor of the experimental group. The findings regarding the corrected post-test mean scores of the experimental and control groups are given in Table 4.

**TABLE 4:** Findings regarding the corrected post-test mean scores of the experimental and control groups.

OCD total score	Group	n	Mean	Corrected mean
Posttest	Experimental	10	18.00	16.768
	Control	13	48.38	50.009

Following the findings in Table 4, the analysis was continued by keeping the pre-test scores under control to obtain information on whether there was a significant difference between the corrected annotated values. As a result of the related analysis, it was concluded that the CBT-based OCD psychoeducation practice applied to adolescents provided significant reductions in the total OCD symptom scores of the participants in the experimental group. Table 5 shows the results of the analysis regarding the total scores of OCD symptoms. Because the research was conducted with a mixed-methods research design, the findings were integrated by giving quantitative and qualitative data together.

**TABLE 5:** Analysis results of OCD symptoms total score.

Source	Sum of squares	*SD*	Mean squares	*F*	*p*	η^2^
Corrected model	6563.383	2	3281.691	23.292	0.000	700
Intercept	277.038	1	277.038	1.966	176	090
OCD total pretest	1345.155	1	1345.155	9.547	006	323
Group	6073.115	1	6073.115	43.104	0.000	683
Error	2817.922	20	140.896			
Total	37837.000	23				
Total error	9381.304	22				

In Table 5, the CBT-based OCD psychoeducation application applied to adolescents resulted in a significant decrease in *OCD symptoms total scores* of the participants in the experimental group [*F*(1, 22) = 9.547, *p* < 0.05, η^2^ = 0.323]. The results of the Bonferroni test also showed that the results of the post-test were in favor of the experimental group.

The qualitative findings obtained through the session evaluation protocol and interviews during and after the experimental application explain and support the result of the experimental process. In the analysis of the documents collected from the participants during the sessions, the statement of **P2** with high OCD symptom levels *“I realized how to deal with my obsessions”* and the statement of **P6** *“I was doing like when I called my father, I was also calling my mother and I was getting the situation approved. I don’t do it now, for example, sometimes I forget to even call my father”* and the statement of **P1** *“I was thinking that I could overcome my goals and obsessions, many of them went away, I overcame some things after the sessions were over”* in the findings obtained as a result of the interview after the experimental application indicate that the program reached the targets set for improvements in OCD symptoms and functionality.

## Discussion

This study aimed to develop a CBT-based psychoeducation practice to reduce OCD symptom levels in a Turkish adolescent sample and to test its effectiveness with a mixed methods research. In the process of developing the intervention program and examining its effectiveness, data triangulation was used. Within the scope of the process, which started with a qualitatively guided approach, an intervention program was developed with document review and analysis. Quantitative and qualitative data collection processes were carried out in order to test the effectiveness of the developed intervention application. Finally, all the findings were interpreted and integrated.

The findings obtained from the quantitative dimension of the study showed that the participants’ scores on the sub-dimensions of doubting-checking, obsessing, hoarding, washing, and ordering were not effective in the sub-dimensions of mental neutralizing, and qualitative findings also supported this finding. It is thought that the reductions in *doubting-checking* symptoms in the study were caused by the participants’ reorganization with interventions based on CBT techniques for the feeling of uncertainty, decision-making processes, and the need for certainty. Cognitive restructuring, procrastination exercises, and response prevention content of the activities designed during the program seemed to enable the participants to reevaluate their doubting-checking symptoms. In the study of Torp et al. (2015), in which they investigated the effect of CBT on OCD symptoms in children and adolescents, the significant reduction in OCD symptoms at the end of a 14-week therapy period including exposure and response prevention, CBT techniques and psychoeducation and also Højgaard et al. (2017)’s conclusion that at the end of the therapy process including CBT and exposure response prevention techniques, children and adolescents achieved significant reductions in OCD symptoms and these gains were maintained after a 1-year follow-up, which supports the findings of the current study.

Perfectionist beliefs about the *obsessing* dimension and strict desires for something to be “complete” and “right” were observed in participants with high obsession symptoms, suggesting that these thought mechanisms may play an active role in obsessions. Coles and Ravid (2016) stated that OCD’s desire to do something “completely” and “right” to prevent harm and reduce anxiety can intensify one’s obsessions and that the reduction in these feelings as a result of CBT makes the person feel less distressed and the fact that Pinto et al. (2017) stated that perfectionism has an important role in the nature and maintenance of OCD and that reductions in the perfectionist thoughts of people with OCD with interventions including CBT techniques support the emphasis in this study on the importance of the perfectionist content of the obsessions. In the interviews about why the participants with high *hoarding* symptoms hoard, it was seen that their beliefs that hoarding is a good action, their development of emotional attachment and attribution meaning to objects, their need to have control over them, and their beliefs that hoarding is a good action are in the foreground. Intervention of these belief and emotional processes of the participants with both alternative thoughts and exposure studies in the restructuring sessions seems to have reduced the hoarding symptoms. As a matter of fact, Williams and Viscusi (2016) stated in their study that excessive emotional attachment to items is one of the important criteria in the evaluation of hoarding, and they stated that emphasizing emotional processes in the treatment process has an important place. In addition, Morris et al. (2016) emphasize the importance of insight level in the severity of hoarding symptoms and treatment response, which supports the fact that working with the concepts discussed in the intervention process is an appropriate initiative.

The most striking aspects of the participants with high *washing* symptoms during the research process are that they experience intense disgust and fear due to the thought of pollution, and as a result of this disgust, they perform repetitive compulsive washing rituals due to the need for security. In this study, it is thought that emotion regulation and cognitive interventions related to the feeling of disgust in washing symptoms have positive effects on compulsive cleaning actions and that the examining thought records techniques of CBT increase cognitive flexibility and provide benefits in the context of preparation in exposure therapies. Indeed, Athey et al. (2015) stated in their research that intense feelings of disgust have an important place in washing symptoms and that the treatment strategy with intensified exposure reduced the feelings of disgust in the participants, which supports our findings. In the study, it was observed that the participant with a high *ordering* symptom score had compulsive ordering attempts stemming from the dysfunctional thought of doing “complete” and “right,” and it was determined that exposure to thought records analysis techniques and exposure ladders, which are among the intervention strategies for these symptoms, reduced symptom scores. As a matter of fact, Taylor (2017) stated that the perceptions of perfectionism regarding cognitive processes in the symmetry/order dimension of OCD and the feeling of lack are effective in the continuation of the symptoms, which supports the concepts discussed in the research.

There were no significant differences in the post-test scores of the experimental and control groups in the findings obtained from the quantitative processes of the study regarding *neutralizing* dimension, one of the OCD symptoms. There may be some reasons why the psychoeducational program regarding the neutralizing dimension could not reach its goal during the research process. One of these is the possibility that the *Child Version of OCI*, used in the process to determine neutralizing symptom levels and monitor the changes, did not fully meet the diversity of neutralizing symptoms (Foa et al., 2010). Another reason is that the participants may have evaluated the neutralizing attempts within the other symptom dimensions of OCD (Clark and Purdon, 2016; Salkovskis and Millar, 2016) and another reason is that the participants may have evaluated the neutralizing symptoms as a temporary, relaxing, and functional action, and this is because actions related to neutralization may have been less studied during intervention studies. Therefore, Ahern et al. (2015) state that neutralizing is less disturbing than other OCD symptoms, and it is perceived as a positive behavior as it can reduce distress instantly in the short term, which supports our findings.

## Conclusion

In conclusion, this study showed that the psychoeducation program developed on the basis of CBT was effective in reducing OCD symptoms in Turkish adolescents, except for the neutralizing dimension. Considering that the psychoeducation program does not provide significant reductions in the neutralizing dimension, which is one of the OCD symptoms, it will be important to plan additional activities related to the neutralizing dimension in future studies to reduce OCD symptoms. In addition, conducting more research that can reveal the clinical features of OCD symptoms in children and adolescents will provide supportive information for preventive and intervention studies to be planned in schools.

### Limitations of the Research

The fact that the experimental implementation process of the research was conducted by the individual who developed the psychoeducation program can be considered as a limitation. In this sense, it can be suggested for future studies that the individual who developed the program should be in the process as an observer instead of being a practitioner at the same time, and another expert should take on the role of practitioner. The fact that the data of the study was obtained from high school students studying in two schools in a province should also be considered in the generalization of the results. In this sense, conducting the process simultaneously in more than one province where different samples can be determined for future research will be an important step in terms of generalizing relevant results. It can be considered as a limitation that only students receive feedback on the changes observed in OCD symptoms in the study. Choosing a mixed approach that includes the opinions of parents and teachers as well as the feedback received from the students may offer a wider evaluation opportunity. Another limitation is that the study was conducted only with adolescents in the Turkish sample. Implementation of the relevant intervention program for adolescents with high OCD symptoms in different countries can make important contributions to the literature in terms of the generalizability of its effectiveness.

## Author’s Note

This article was produced from MŞ’s doctoral dissertation completed in 2020 (Thesis No: 657089).

## Data Availability Statement

The original contributions presented in the study are included in the article/supplementary material, further inquiries can be directed to the corresponding author/s.

## Ethics Statement

The studies involving human participants were reviewed and approved by the Atatürk University Educational Sciences Ethics Committee. Written informed consent to participate in this study was provided by the participants’ legal guardian/next of kin. Written informed consent was obtained from the minor(s)’ legal guardian/next of kin for the publication of any potentially identifiable images or data included in this article.

## Author Contributions

MŞ and İS: developing the research idea, completing the research procedures, creating online data collection processes and delivering them to the target groups, transfer of the data collected to the SPSS environment, writing the manuscript. MŞ: conducted the data collection and analysis. All authors contributed to the article and approved the submitted version.

## Conflict of Interest

The authors declare that the research was conducted in the absence of any commercial or financial relationships that could be construed as a potential conflict of interest.

## Publisher’s Note

All claims expressed in this article are solely those of the authors and do not necessarily represent those of their affiliated organizations, or those of the publisher, the editors and the reviewers. Any product that may be evaluated in this article, or claim that may be made by its manufacturer, is not guaranteed or endorsed by the publisher.
